# Similar Energy Expenditure During BodyPump and Heavy Load Resistance Exercise in Overweight Women

**DOI:** 10.3389/fphys.2020.00570

**Published:** 2020-06-10

**Authors:** Anne Mette Rustaden, Christina Gjestvang, Kari Bø, Lene Annette Hagen Haakstad, Gøran Paulsen

**Affiliations:** Department of Sports Medicine, Norwegian School of Sport Sciences, Oslo, Norway

**Keywords:** resting metabolic rate, RMR, EPOC, group exercise, energy consumption

## Abstract

**Purpose:**

High-repetition, low-load resistance exercise in group class settings has gained popularity in recent years, with BodyPump as a prime example. For individuals using exercise for body-weight management, the energy expenditure during exercise is of interest. Therefore, we herein aimed to estimate the energy expenditure during a session of BodyPump and a time-matched session of heavy load resistance training in overweight women (BMI ≥ 25.0).

**Methods:**

Eighteen women participated in the study (mean age 35.4 years ± 10.2, BMI 30.4 kg/m^2^ ± 4.8), 10 exercising BodyPump (50–100 repetitions each muscle group) and eight performed a heavy load session (eight repetition maximum × three sets). The energy expenditure was assessed with indirect calorimetry during the sessions and for two intervals at rest during the recovery phase: 0–20 and 120–140 min after the sessions.

**Results:**

The BodyPump group lifted significantly more loads than the heavy load group (19,485 kg ± 2258 vs 15,616 kg ± 2976, *p* = 0.006), while energy expenditure was similar with 302 kcal ± 67 and 289 kcal ± 69 in BodyPump and heavy load group, respectively (*p* = 0.69). With no group differences, the resting metabolic rate (RMR) was elevated with 15–22% 2 h after exercise.

**Conclusion:**

Overweight women achieved an energy expenditure of approximately 300 kcal (4.7 kcal per min) during a single session of BodyPump, which was similar with the women performing a single session of heavy load resistance exercise.

## Introduction

The worldwide prevalence of overweight (BMI ≥ 25.0 kg/m^2^) and obesity (BMI ≥ 30.0 kg/m^2^) have increased considerably during the last three decades (Ng et al., 2014; Di Cesare et al., 2016). According to the World Health Organization, 40% of the adult female population are classified as overweight and 15% as obese [World Health Organization (WHO), 2020]. Several lifestyle related interventions have been investigated to treat overweight and obesity and prevent weight gain, and today a combination of energy restrictions, physical activity, and behavioral change strategies are recommended (Donnelly et al., 2009; Laddu et al., 2011; Dombrowski et al., 2014; Samdal et al., 2017). Traditionally, endurance training have been prioritized as physical activity among overweight and obese, but the American College of Sports Medicine (ACSM) also recommend adults to perform regular resistance exercise [two to three times/week with an intensity between 60 and 80% of one repetition maximum (1RM)] (Garber et al., 2011).

Increased energy expenditure is imperative if exercise is used to reduce body weight through loss of fat mass; however, traditional resistance exercise does not appreciably elevate energy expenditure relative to other exercise modalities (e.g., endurance training) (Donnelly et al., 2009; Willis et al., 2012; Swift et al., 2018). Despite this knowledge, health- and fitness clubs offer different resistance-exercise-based group classes and claim these to be effective in improving body composition and reduce body weight. A strategy used to augment the energy expenditure in many of these classes is to increase the worktime to rest ratio, i.e., apply a high duty cycle (work time divided by total exercise time). This translates into resistance exercise modes with low-loads, high number of repetitions, and short rest-intervals between sets and exercises (Stanforth et al., 2000; Rixon et al., 2006; Berthiaume et al., 2015; Harris et al., 2018).

BodyPump is the most popular resistance-exercise-based group class worldwide, available in almost 15,000 health- and fitness clubs (Les Mills International). The distributor, Les Mills International (2020), pre-choreograph the classes, all based on the same principle; a full body-workout with barbell and weights and a high duty cycle: 800–1000 repetitions per session/h, low loads (<35% of 1RM) (Rustaden et al., 2017) and short rest-intervals (<20 s). According to LesMills, this formula results in a high energy expenditure (up to 540 kcal each BodyPump session^1^).

Previously, the energy expenditure during a session of BodyPump has been assessed in young, normal weight, and trained men and women (Stanforth et al., 2000; Berthiaume et al., 2015; Harris et al., 2018). In these studies, the energy expenditure was reported to be 250–334 kcal each session. However, only Stanforth et al. (2000) measured the actual oxygen consumption during the exercise session. Berthiaume et al. (2015) and Harris et al. (2018) only estimated the energy expended based on a movement sensor (SenseWear armband) and heart rate (HR), respectively. Moreover, changes in resting metabolic rate (RMR) after the exercise session—excess post exercise oxygen consumption (EPOC)—was not assessed in any of these studies. The magnitude of EPOC seems to be positively dependent on the work done during exercise (intensity and duration) and may have relevance in body-weight management (Borsheim and Bahr, 2003).

Given that BodyPump is globally popular and available in fitness centers worldwide, it is valuable to gain knowledge of the physiological responses to this exercise mode; not the least, insight in the energy expenditure for individuals that exercise for body-weight management. Therefore, the aim of the present study was to assess the energy expenditure from BodyPump in middle-aged, overweight women. In addition, the BodyPump session was compared to a time-matched session of traditional heavy load resistance training in accordance with the ACSM recommendations (Garber et al., 2011). We hypothesized that the energy expenditure would be higher during and after BodyPump than a heavy load resistance exercise session, because BodyPump has a high duty cycle and should result in a larger total work (repetitions times load).

## Materials and Methods

### Study Design and Participants

Participants were recruited from an on-going randomized controlled trial (RCT), where overweight and obese women were randomized to either BodyPump, heavy load resistance exercise (with or without a personal trainer), or non-exercising controls (for further details, see Rustaden et al., 2017). Inclusion criteria were BMI ≥ 25.0, age 18–65, and being untrained defined as “not performing regular structured exercise ≥ twice a week for the last 6 months.”

The main aims for the RCT study were to investigate the effects of 12 weeks of these exercise interventions on muscle strength and body composition. In the present study, we wanted to investigate the energy expenditure of a single session of BodyPump and a single session of heavy load resistance exercise. All participants in the BodyPump and personal trainer group were invited to participate in the present study.

In total, 18 women volunteered to participate in the present study (mean age 36 years ± 10, weight 84 kg ± 14, height 168 cm ± 6, and BMI 30 kg/m^2^ ± 5), 10 from the BodyPump group and eight from the heavy load resistance exercise group. The women were initially untrained but had, at the point of testing in the present study, been training either BodyPump or heavy load resistance exercise for 3–4 weeks (9–12 sessions). All participants were therefore well accustomed to the exercise routines when entering the present, acute study. In the present study, those who were part of the BodyPump group in the RCT conducted the BodyPump session, while those who were in the heavy resistance exercise group in the RCT, conducted the heavy resistance exercise session.

In the present study, we aimed to estimate the energy expenditure from a BodyPump and a heavy load resistance exercise session. In brief, the participants conducted an exercise session, BodyPump or heavy load resistance exercise, while the oxygen uptake was measured continuously. The oxygen uptake was used to estimate the energy expenditure during the exercise sessions (Compher et al., 2006). EPOC was assessed by measuring oxygen uptake at rest, i.e., RMR, conducted before and twice after the exercise sessions. The time slots for the RMR assessments were chosen based on similar, previous studies (Binzen et al., 2001; Benton et al., 2016). Total work (load × repetitions × sets) and HR were also assessed during the exercise sessions.

The participants arrived at the laboratory after 12 h fast. Caffeine and nicotine were prohibited before testing, and the participants were instructed to use car or public transportations to the laboratory. Strenuous physical activity or exercise was asked to be avoided 48 h before the test day. The test day was initiated by RMR measurements between 7:45 and 8:30 am, followed by a standard breakfast (oeatmeal) with a caloric content equivalent to 20% of the individual’s estimated RMR (between 8:30 and 9:00 am). The exercise sessions occurred between 9:00 and 10:00 am. After the exercise sessions, RMR was assessed for 20 min. The women were thereafter given a standardized lunch (same as the breakfast; at 10:30 am) and they rested in a seated position before the final RMR measurement 2 h after exercise (120–140 min). In total, the testing procedure lasted for approximately 4 h.

### Ethics Statement

The study is approved by the Regional Committee for Medical Research Ethics Norway, Oslo (REK 2012/783). All participants signed a written consent statement before entering the study, and the procedures followed the World Medical Association Declaration of Helsinki.

### Assessments

#### Energy Expenditure

Energy expenditure was assessed by indirect calorimetry, applying an automatic ergospirometry system (Oxycon Pro Jaeger Instrument, Hoechberg, Germany). The participants breathed through a Hans Rudolph mask attached to a 3-m long non-rebreathing hose, allowing the participants to move freely during the exercises (an investigator manually assisted by positioning/controlling the hose). The measurements started 2 min before exercise and continued during the entire exercise sessions. O_2_ and CO_2_ were continuously sampled (in a mixing chamber) and averaged over 30 s periods. Prior to each test, the Oxycon Pro Jaeger Instrument was calibrated after the manufacturers’ guidelines. Indirect calorimetry is a valid assessment method when estimating energy expenditure (Compher et al., 2006), and the Oxycon Pro Jaeger Instrument system applied has been found to be a highly accurate and valid system (Foss and Hallèn, 2005).

The energy expenditure (kcal) was calculated as the accumulated O_2_ consumption during exercise multiplied by 5 kcal (McArdle et al., 2010).

#### Resting Metabolic Rate

Resting metabolic rate was estimated by indirect calorimetry with a ventilated hood (Canopy-option for Oxycon Pro Instrument). The participants were in supine position on a comfortable bed, the test lab was quiet, had dimmed light, and the temperature was 22–24°C. The measurements lasted for 30 min, but the initial 10 min was discarded (Compher et al., 2006). The calorie equivalent used to estimate energy expenditure was derived from each participant’s respiratory exchange ratio (RER) and ranged from 4.68 to 5.04 kcal per liter oxygen (LO_2_) (McArdle et al., 2010). The energy expenditure was calculated as calories each minute = VO_2_ (Lmin^–1^) × kcal per LO_2_.

#### Total Workload

The work done during exercise was calculated by multiplying the load used in each exercise (kg) by the repetitions and sets for each participant. The body mass was included as load in exercises where the center of mass was moving vertically: In squats and lunges 90% of the body mass was added to the external load (e.g., 80 kg × 0.9 + 30 kg = 102 kg). In push-ups, dips, and sit-ups, 65, 50, and 40% of the body mass were used, respectively. These estimations were based on pilot testing on a force plate (AMTI, SG-9, Advanced Mechanical Technologies, Newton, MA, United States).

#### Heart Rate

Heart rate was registered by using an HR monitor (Polar RS800, Kempele, Finland) during the exercise sessions. Maximal HR was estimated: 211 – 0.64 × age (Nes et al., 2013).

### Exercise Protocols

The participants conducted either a session of BodyPump (Table 1) or heavy load resistance exercise (Table 2). A personal trainer was present during all sessions to ensure proper lifting technique and assist if necessary, but did not interfere with the exercise protocol.

**TABLE 1:** Description of BodyPump release no. 83, with exercises and number of repetitions.

Music no.	Exercise	Volume (reps)
1 Warming-up	Straight leg deadlift, rowing, shoulder press, squat, lunges, and biceps curl	88
2 Leg	Squat	95
3 Chest	Bench press	80
4 Back	Rowing, stiff legged deadlift, clean and press, and power press	75
5 Triceps	French press, triceps press, pullover, and overhead triceps press	78
6 Biceps	Biceps curl	68
7 Leg	Squat, lunges, and squat jump	72 + 24 jumps
8 Shoulders	Push-up, lateral raise, rowing, and shoulder press	76 + 36 push up
9 Abdominals	Sit-ups, sit-ups to the side, and side-plank	51 + 30 s

**TABLE 2:** Description of the heavy load resistance exercise program, showed with exercises and training volume.

Exercise	Volume (sets × reps)
Squat	3 × 8
Lunges	4 × 8
Stiff-legged deadlift	3 × 8
Forward rowing	3 × 8
Bench press	3 × 8
Dips	2 × 8
Shoulder press	2 × 8
Lateral raise	2 × 8
Clean and press	2 × 8
Triceps press overhead	2 × 8
Biceps curl	2 × 8
Sit-ups	3 × 8

#### BodyPump

BodyPump is a high-repetition low-to moderate group session, prechoreographed and distributed by LesMills International. Every third month LesMills releases a new BodyPump program, all based on the same model and principles (LesMills International). During the intervention period in the RCT study, BodyPump release no. 83 was present at all health- and fitness clubs worldwide, including nine music tracks (4–7 min), each exercising specific body parts. All the 1-h sessions includes approximately 800 repetitions in total, and 50–100 repetitions in each muscle group. The participants exercise with a step and free-weights (1, 2.5, or 5 kg), which they put together on a 1.25 kg bar. Between each track, there is a short rest period of approximately 1 min, used to change weights and prepare to the next exercises. Some of the tracks include short inter-session rest periods (typically 16–32 beats and 7–14 s) (LesMills International). During the assessment in the present study, participants were instructed from a LesMills video Les Mills International (2020), with an instructor demonstrating the whole session. As mentioned above, the participants were familiar with the exercise program, as assessments were conducted midway into the RCT study. The external loads for each exercise used were based on the instructions, and the participants experience. The video-instructor encouraged the participants to achieve muscular fatigue in each track, with proper lifting technique.

#### Heavy Load Resistance Exercise

The heavy load resistance exercise group performed session 1 from week 5–8 in the RCT (Rustaden et al., 2017), including 8RM × two to four sets, and 45 and 60 s of rest between sets and the exercises, respectively (Table 2). The participants selected the exercise loads based on their experience and, if necessary, with assistance from the personal trainer overseeing the sessions. It was important that the loads were as heavy as possible for the eight repetitions per set.

### Statistical Analysis

Data are presented as means with standard deviation (±) for all variables. A normal distribution of the data was found using a Shapiro–Wilk test, and an independent *t*-test was used to compare between-group differences in total workload and energy expenditure during the sessions. A mixed between-within subject’s analysis of variance assessed the impact of the two different exercise programs on O_2_ ml/kg, RMR (20 min), HR (beats/min), and RER at the three assessment time points. Analyses were conducted with SPSS Statistical Software version 21 (IBM Corporation, Route, Somers, NY, United States). Level of significance was *p* ≤ 0.05.

## Results

There were no significant differences in demographic variables between the two experimental groups (Table 3). The duty cycles (active time during the sessions) were 86 and 45% for BodyPump and heavy load resistance exercise, respectively.

**TABLE 3:** Demographic data of all participants in the BodyPump group and the heavy load resistance exercise group (RE).

Variable	BodyPump (*n* = 10)	RE (*n* = 8)	*p*-value
Age (year)	36.4 ± 9.9	34.1 ± 11.0	0.651
Weight (kg)	84.7 ± 13.5	87.1 ± 16.4	0.744
Height (cm)	167.1 ± 6.6	168.9 ± 6.7	0.562
BMI (kg/m^2^)	30.3 ± 4.7	30.5 ± 5.3	0.967
Fat mass (%)	38.1 ± 7.4	38.6 ± 5.2	0.275
Muscle mass (kg)	28.8 ± 3.2	30.4 ± 3.6	0.270

*Presented as mean with standard deviation (±) and differences between groups with *p-*value.*

### Energy Expenditure

The estimated total energy expenditure during exercise was not significant different between the groups (*p* = 0.696) with 302 kcal (±67) during BodyPump and 289 kcal (±69) during heavy load resistance exercise (Table 4). The individual range was 170–378 kcal in BodyPump and 169–347 kcal in the heavy load resistance exercise group.

**TABLE 4:** Exercise duration (min/session), oxygen uptake (O_2_), respiratory exchange ratio (RER), heart rate, kilocalories (kcal) each minute and total energy expenditure in the BodyPump, and heavy load resistance exercise group (RE).

Variable	BodyPump (*n* = 10)	RE (*n* = 8)	*p*-value
Duration [min (session)]	53.0 ± 0.0	57.7 ± 2.9	0.033*
O_2_ (ml/min/kg)	12.3 ± 2.7	12 ± 2.0	0.779
RER	0.96 ± 0.0	0.94 ± 0.0	0.373
Heart rate (beats/min)	142 ± 16	146 ± 13	0.592
Kcal/min	4.7 ± 1.2	4.0 ± 1.0	0.200
Total energy expenditure (kcal)	302 ± 67	289 ± 69	0.696

*Indicates a significant difference between the groups with *p* < 0.05. Presented as mean with standard deviation (±) and *p*-value showing group differences.

### Resting Metabolic Rate

There were no statistically significant differences between the exercise modalities in RMR 0-20 or 120–140 min after exercise (Table 5). Oxygen uptake (O_2_ ml/min), RER, RMR, and HR were assessed at supine rest for 20 min before exercise, immediately after (0–20 min) and 120–140 min after exercise. The mixed between-within subject’s analysis of variance revealed no significant interaction effect between the groups. In both groups, there was a significant effect for time (*p* < 0.005), but the main effect comparing the two groups was not significant (Table 5). In the BodyPump group, RMR increased 29% from before exercise to immediately after exercise, and 22% from before exercise to 2 h after exercise (*p* < 0.001). For the heavy load resistance exercise group changes in RMR were 33 and 15% before to immediately after, and before to 2 h after exercise, respectively (*p* < 0.001).

**TABLE 5:** Variables from resting metabolic rate (RMR) before exercise, after exercise acute (0–20 min), and after exercise 2 h (120–140 min) in the BodyPump and heavy load resistance exercise group (RE).

Variable	Before exercise		After exercise 0–20 min		After exercise 120–140 min		Interaction	Time	Between groups
	BodyPump	RE	BodyPump	RE	BodyPump	RE	*p-*value (pes)	*p*-value (pes)	*p*-value (pes)
O_2_ ml/kg	2.3 ± 0.4	2.6 ± 0.4	3.0 ± 0.5	3.4 ± 0.5	3.0 ± 0.4	3.0 ± 0.7	0.240 (0.17)	<0.0005* (0.75)	0.336 (0.06)
RER	0.87 ± 0.13	0.77 ± 0.03	0.78 ± 0.04	0.75 ± 0.04	0.84 ± 0.05	0.81 ± 0.07	0.473 (0.11)	<0.0005* (0.70)	0.076 (0.21)
RMR kcal	19 ± 4	21 ± 2	25 ± 4	29 ± 4	24 ± 4	25 ± 5	0.194 (0.20)	<0.0005* (0.78)	0.181 (0.11)
HR (beats/min)	65 ± 8	66 ± 9	89 ± 10	93 ± 8	72 ± 10	72 ± 11	0.474 (0.10)	<0.0005* (0.96)	0.751 (0.01)

*Indicates a significant difference in time within both groups with *p* < 0.05. Presented with mean values and standard deviation (±). Interaction between the groups, substantial main effect for time in the two groups, and main effect comparing the two groups are shown with *p*-value and partial eta squared (pes).

### Total Workload

Including both external loads and part of the body mass, the BodyPump group lifted significantly more loads (19,485 kg ± 2258) than the heavy load resistance exercise group (15,616 kg ± 2976) (*p* = 0.006). Load lifted per minute was also significantly higher in BodyPump compared to the heavy load resistance exercise group (*p* = 0.001), with 368 kg/min (±43) and 280 kg/min (±50), respectively. Based on the participants’ 1RM tests at baseline in the RCT study (Rustaden et al., 2017), the relative loads (% of 1RM) in the BodyPump group were estimated to 14% (±2.8) and 18% (±2.6) in squat and bench press, respectively. The relative loads in the heavy load resistance exercise group were 77% (±16.5) in squat and 80% (±8.0) in bench press, which were significantly higher than the BodyPump group (both *p* ≤ 0.001).

## Discussion

To our knowledge, this is the first study to compare the energy expenditure during BodyPump session with traditional heavy load resistance exercise in overweight women. We observed that the BodyPump participants performed more work (kg lifted) than the heavy load resistance exercise group. Nevertheless, energy expenditure during the workouts were about 300 kcal and similar between the two exercise modalities. The RMR was elevated for at least 2 h after exercise, with no differences between BodyPump and heavy load resistance exercise; i.e., the EPOC appeared similar between sessions.

The higher total workload performed in BodyPump, compared to the heavy load resistance exercise, was due to the higher number of repetitions, as well as fewer and shorter periods of rest. The BodyPump program included approximately 800 repetitions, and 10 min of rest in total. In comparison, the heavy load resistance exercise program included 248 repetitions, and approximately 28 min of rest. Thus, the heavy load resistance exercise group had a higher energy expenditure per kg lifted. It is also likely that the exercises in this group were performed with larger range of motions, compared to BodyPump, as they used 2–4 s per repetition. In BodyPump, the participants had to keep up with the choreography and music, which is approximately one lift each second. This faster lifting pace in BodyPump might have resulted in smaller range of motions, and consequently, less energy used per repetition.

In correspondence with the VO_2_-measurements, mean HR was similar between the two exercise modalities (142 beats/min in BodyPump and 146 beats/min in the heavy load resistance exercise group). The estimated relative intensities (HR_max_) were 76 and 77% in BodyPump and heavy load resistance exercise, respectively, which indicate a similar cardiovascular load. This correspond with Oliveira et al. (2009), who investigated the physiological profile during a BodyPump session, and found HR_max_ to be 78 and 84%, during the tracks involving the largest muscle groups (Oliveira et al., 2009).

Total energy expenditure during BodyPump was somewhat higher in the present study, compared to previous findings. Stanforth et al. (2000) and Berthiaume et al. (2015) investigated physiological responses during a BodyPump session in 30 and 40 trained men and women, respectively. Total energy expenditure in Stanforth et al. (2000) was 265 kcal (±60) including both genders, and for women only; 214 kcal (±26). Berthiaume et al. (2015) reported 250 kcal (±68) in both genders, and 202 kcal (±38) in the female participants (assessed with SenseWear armband, not O_2_ uptake). Higher body mass in our participants could explain the discrepancy in energy expenditure, compared to these two studies. Since body mass makes up most of the load in exercises such as squats and lunges, our overweight participants probably used more energy per repetition as they were about 23 kg heavier than the normal weight women in Stanforth et al. (2000). Berthiaume et al. (2015) did not report the participants body weight, but mean BMI in their female participants were 22.7 kg/m^2^ (±2.2), compared to 30.3 kg/m^2^ (±4.7) in our participants exercising BodyPump. In addition, the assumption is further supported by differences in exercise intensity. Our women exercised with a relative intensity of 76% of HR max (mean 142 beats/min) compared to 63% of HR max (mean 124 beats/min) in the Stanforth et al. (2000) study. HR was not reported in Berthiaume et al. (2015). Different assessment methods may also explain some of the differences in exercise intensity. On the other hand, estimated energy expenditure in the present study is comparable with Harris et al. (2018), with 334 kcal in female participants with minimum 6 months of Body Pump experience. Anyhow, the findings of the present and previous studies indicate that the energy expenditure is significantly less than the energy costs (540 kcal) claimed by LesMills (LesMills International). Interestingly, Berthiaume et al. (2015) asked their participants of perceived energy expenditure after the session, and both men and women overestimated the measured energy expenditure by 50 and 84%, respectively. This emphasizes the importance of informing the public about realistic expectations to energy expenditure during exercise modes as BodyPump. In perspective, 4.7 kcal/min energy expenditure, as seen during the BodyPump session, equals a comfortable walking speed on a flat surface (3–4 km/h for an ∼84 kg person (McArdle et al., 2010).

Rixon et al. (2006) investigated energy expenditure in normal weighed women during four different group-based exercise concepts, and found that 60 min of bodycombat, step-aerobics, indoor-cycling, and aerobic pump with resistance exercises resulted in 8–10 kcal/min and moderate to high intensity (55–89% of HR_max_) (Rixon et al., 2006). Compared to Rixon et al. (2006), both groups in the present study exercised with merely half of the energy expenditure per unit time (BodyPump group 4.7 kcal/min ± 1.2, and heavy load resistance exercise group 4.0 kcal/min ± 1.0). Furthermore, according to the ACSM position stand (Donnelly et al., 2009), physical activity with moderate intensity, resulting in energy costs between 1200 and 2000 kcal/week is recommended to prevent weight gain or give a moderate weight loss (Donnelly et al., 2009). Based on observations in the current study, a minimum of four weekly sessions of BodyPump or heavy load resistance exercise would contribute to achieving this recommendation. In fact, in two other studies from our study group, which the current participants were part of; body composition was unchanged after 12 weeks of BodyPump (Rustaden et al., 2017, 2018). In other words, the energy expenditure of each BodyPump session (or heavy load resistance exercise session) was too small to cause a loss in body mass.

The present results indicate elevated RMR due to EPOC after both BodyPump and heavy load resistance exercise with values in line with similar studies (Borsheim and Bahr, 2003; LaForgia et al., 2006). Based on similar changes in BodyPump and heavy load resistance exercise, we suggest that the EPOC was more related to the cardiovascular load and muscular energy turnover than the mechanical loading (i.e., differences in external loads/weights). As concluded by several authors, the magnitude and duration of EPOC after exercise seem highly correlated to cardiovascular intensity, expressed as % of HR_max_ or % of VO_2__max_ (Borsheim and Bahr, 2003; LaForgia et al., 2006; Paoli et al., 2012). In contrast to our findings, Thornton and Potteiger (2002) found similar acute energy expenditure, but greater EPOC in a high-load resistance exercise group (85% of 8RM) than a low-load resistance exercise group (45% of 8RM). Interestingly, Thornton and Potteiger (2002) reported higher cardiovascular load and muscular energy turnover rates in the high-load group, as judged by HR and blood lactate, respectively. Thus, this could explain the higher EPOC in the high-load group. In our study, BodyPump (low load) compensated for lower loads with a higher duty cycle, and thereby eliciting similar cardiovascular and muscular stress as heavy load resistance exercise. Of note, our participants were well accustomed to the exercises before the test sessions. This contrasts with most other studies on EPOC, where the participants conducted the exercise session for the first time. Unaccustomed resistance exercise may lead to some exercise-induced muscle damage, which again may affect the EPOC values (Thornton and Potteiger, 2002; Borsheim and Bahr, 2003; LaForgia et al., 2006; Hackney et al., 2008; Paoli et al., 2012). This is a weakness in many other studies as exercise-induced muscle damage will decrease drastically after only one session—known as the repeated-bout effect (McHuge, 2003); thus, many of the EPOC values reported after resistance exercise may be an overestimate of the EPOC that can be expected after exercise sessions regularly repeated in a training period.

This study has several weaknesses. A better design to compare energy expenditure might have been a cross-over design where each individual had conducted both exercise sessions. However, since the participants were recruited from an ongoing RCT study, we could not interfere with the interventions. In addition, the study may have a small sample size, which could lead to type II error. Another limitation of the study is that EPOC was still present during our last assessment period (2 h after exercise), meaning that we did not capture the duration and total magnitude of EPOC. We can therefore not be sure there were no group differences at later time-points. Furthermore, we did not include a control day without exercise. Thus, we cannot claim that the EPOC assessed was only due to the exercise sessions. The RMR after exercise may have been affected by the two light meals and time of day (Borsheim and Bahr, 2003). However, Haugen et al. (2003) found that repeated morning and evening assessments of RMR were stable and highly correlated with only 6% variability. Thus, these design weaknesses should not interfere with the main purpose with the present study, to compare BodyPump against heavy load resistance exercise.

## Conclusion

A single session of BodyPump resulted in an energy expenditure of approximately 300 kcal (or 4.1–4.7 kcal/min), which was similar as the energy expenditure from a session of heavy load resistance exercise. An energy expenditure of approximately 300 kcal during an 1 h session are considered as low to moderate, and comparable to the energy expenditure from brisk walking. Similar levels of EPOC were observed after the two exercise sessions.

## Data Availability Statement

The datasets generated for this study are available on request to the corresponding author.

## Ethics Statement

The studies involving human participants were reviewed and approved by the Regional Committee for Medical Research Ethics Norway, Oslo. The patients/participants provided their written informed consent to participate in this study.

## Author Contributions

All authors have been included in the planning of the study and the writing procedure. AR had the main responsibility of the whole study and was the main writer. CG was responsible for the assessments and been involved in the writing. KB and LH were the supervisors and involved in the writing. GP was the main supervisor and involved in the analyses and the writing.

## Conflict of Interest

The authors declare that the research was conducted in the absence of any commercial or financial relationships that could be construed as a potential conflict of interest.

## Footnotes

1 https://www.lesmills.com/workouts/fitness-classes/bodypump, April 2020.
